# Compression of the Aorta in Uterine Hemorrhage

**Published:** 1850-01

**Authors:** Robt. Crane

**Affiliations:** Middlebury, Conn.


### ARTICLE V.

*Compression of the Aorta in Uterine Hemmorrhage.*—(Communicated for the Boston Medical and and Surgical Journal.)

The application of arterial pressure to arrest formidable uterine hemorrhage, is not presented here as a novelty. The merit of its introduction is probably due to the veteran Baudelocque; after him, it was adopted and recommended by Chailly; while the practice has been further confirmed by cases presented to to the notice of the profession by Mr. Pretty, J. D, Brown, and many others. Still its adoption has not been in proportion to its merits; and in circumstances where it might have afforded timely succor, doubtful and hazardous experiments have often been resorted to, attended with confusion to the accoucheur and peril to the patient. It has been my reliance in numerous instances during the past six years, and with so happy results, that I have come to regard any degree of *post-partum* hemorrhage so easily controlled, as to constitute an accident of no very grave moment. It is a resort at once safe, practicable and efficient. Even when the stomach will readily tolerate ergot, and every other ordinary means can be made subservient, there is often an interval before their efficient operation can be obtained, when the patient's life is momentarily endangered by delay. At this critical juncture, compression of the aorta can be brought to bear with signal advantage, while it will not embarrass, but rather assist the ordinary efforts of both nature and art towards a favorable issue. We should by no means neglect the usual appliances at hand; but are at liberty, especially if the services of a reliable assistant are at command, to resort to the application of cold, associated with manual compression of the uterine tumor. By this means the patient's life is placed beyond jeopardy for the instant, and an extension of time is gained, in which to induce that fixed contraction, short of which no attendant could abandon his charge with any degree of intelligent satisfaction and composure.

Neither in such cases should our aim be barely to save life from the extremity of peril. There is a degree of hemorrhage, graduated by individual circumstances, beyond which it should be considered a calamity for our patients to succumb.

The shock to the system produced by extreme depletion, frequently saps the foundations of health and vigor, and opens avenues for the approach of some insidious and deadly mischief.

In relation to the *modus operandi*, the aorta should be compressed in the umbilical region just before its iliac bifurcation. At this point, after the partial descent of the uterus, there is seldom any intervening obstacle; the parietes of the abdomen lie near the spine, and readily yield on account of thei flaccidity; and should any portion of intestine happen to be floating in the way, it readily eludes the touch, and the hand is at once upon the aorta strongly pulsating, and feeling under the finger like a large whip cord. The pulsations can be readily controlled by firm, steady, and not very forcible pressure; and this can be brought to bear with the greatest facility by a thumb and one finger, or any two fingers, so placed in juxtaposition as to bring the triangular space formed at their extremities to fit over the artery like a saddle, and by this means prevent it rolling from the grasp, as it is liable to do without some such precaution.

The demand for this arterial compression will of course be proportioned to the intensity of the hemorrhage and the condition of the patient; but in the event of flooding, however sudden or appaling, I believe the physician has here at ready command the key that may infalliably and safely check the flow of the vital current. ROB'T CRANE, M.D.

*Middlebury, Conn., Nov.* 20, 1849.

